# Interspecific Gene Flow and Selective Sweeps in *Picea wilsonii*, *P. neoveitchii* and *P. likiangensis*

**DOI:** 10.3390/plants11212993

**Published:** 2022-11-06

**Authors:** Yifu Liu, Aili Qin, Ya Wang, Wen Nie, Cancan Tan, Sanping An, Junhui Wang, Ermei Chang, Zeping Jiang, Zirui Jia

**Affiliations:** 1 Key Laboratory of Forest Ecology and Environment of National Forestry and Grassland Administration, Ecology and Nature Conservation Institute, Chinese Academy of Forestry, Beijing 100091, China; 2 State Key Laboratory of Tree Genetics and Breeding, Chinese Academy of Forestry, Beijing 100091, China; 3 Research Institute of Forestry, Chinese Academy of Forestry, Beijing 100091, China; 4 Research Institute of Forestry of Xiaolong Mountain, Gansu Provincial Key Laboratory of Secondary Forest Cultivation, Tianshui 741002, China

**Keywords:** gene flow, selective sweeps, *Picea wilsonii*, *Picea neoveitchii*, *Picea likiangensis*

## Abstract

Genome-wide single nucleotide polymorphism (SNP) markers were obtained by genotyping-by-sequencing (GBS) technology to study the genetic relationships, population structure, gene flow and selective sweeps during species differentiation of *Picea wilsonii*, *P. neoveitchii* and *P. likiangensis* from a genome-wide perspective. We used *P. jezoensis* and *P. pungens* as outgroups, and three evolutionary branches were obtained: *P. likiangensis* was located on one branch, two *P. wilsonii* populations were grouped onto a second branch, and two *P. neoveitchii* populations were grouped onto a third branch. The relationship of *P. wilsonii* with *P. likiangensis* was closer than that with *P. neoveitchii*. ABBA-BABA analysis revealed that the gene flow between *P. neoveitchii* and *P. wilsonii* was greater than that between *P. neoveitchii* and *P. likiangensis*. Compared with the background population of *P. neoveitchii*, the genes that were selected in the *P. wilsonii* population were mainly related to plant stress resistance, stomatal regulation, plant morphology and flowering. The genes selected in the *P. likiangensis* population were mainly related to plant stress resistance, leaf morphology and flowering. Selective sweeps were beneficial for improving the adaptability of spruce species to different habitats as well as to accelerate species differentiation. The frequent gene flow between spruce species makes their evolutionary relationships complicated. Insight into gene flow and selection pressure in spruce species will help us further understand their phylogenetic relationships and provide a scientific basis for their introduction, domestication and genetic improvement.

## 1. Introduction

Spruce (*Picea*) is an important component of coniferous forests in the Northern Hemisphere and is widely distributed there [1]. Most conifers have a long generation time, a large population size [2,3,4], weak interspecific reproductive isolation [5] and slow molecular evolution [6,7]. *Picea* experienced rapid differentiation during the two periods of Qinghai-Tibet Plateau uplift in the Pliocene and the Quaternary glacial climate oscillation [8], and the frequent interspecific gene flow and reticulate evolution within the genus [9] have further complicated interspecific evolutionary relationships. Gene flow refers to the process by which genes move spatially from one location or group to another [10]. Species differentiation can occur despite gene flow [11]; that is, there is continuous gene flow between species with near or sympatric distributions, and new species are generated through differential selection [12,13]. For conifers with long generation times, it can take a long time from the start of differentiation to the cessation of gene flow. It has been suggested that gene flow between different species continues to decrease over time when species are under isolation or selection pressure [14]. Therefore, clarification of gene flow and selection pressure between spruce species is important for further understanding the phylogeny of *Picea*.

Asia, with 24 of the 35 species of spruce [1,15], is one of the hotspots of spruce diversity. Among these species, *P. wilsonii*, *P. neoveitchii* and *P. likiangensis* are endemic to China. *P. wilsonii* and *P. neoveitchii* are distributed in Central and Western China, whereas *P. likiangensis* is distributed in areas with relatively high altitudes in Southwest China. Although *P. likiangensis* and *P. neoveitchii* are heterogeneously distributed, *P. wilsonii* has an overlapping distribution with *P. neoveitchii* and neighbouring distribution with *P. likiangensis*, which provides an opportunity for gene flow between the three species. A phylogenetic tree based on chloroplast DNA (cpDNA) sequences [16] and comprehensive cpDNA sequences, mitochondrial DNA (mtDNA) sequences and nuclear gene fragments [17] showed that *P. wilsonii* and *P. neoveitchii* were all clustered in a clade but were not sister species, while the genetic relationship between *P. wilsonii* and *P. likiangensis* was closer than that between *P. wilsonii* and *P. neoveitchii* [18]. *P. wilsonii* and *P. neoveitchii* differentiated earlier, approximately 16 million years ago (mya) [19], while *P. wilsonii* and *P. likiangensis* differentiated later, at approximately 6.3 mya [20]. Zou et al. [19] hypothesised that *P. morrisonicola* recently diverged from *P. wilsonii* and that there was continuous gene flow based on 18 gene fragments from the chloroplast, mitochondrial and nuclear genomes, while there was also continuous mtDNA gene flow from *P. neoveitchii* to *P. wilsonii*. Wang et al. [21] investigated the degree and direction of gene interpenetration among three spruce species in their sympatric distribution area based on cpDNA, mtDNA and nuclear gene fragments and showed that mtDNA, which has the lowest amount of gene flow, has the highest degree of introgression, with little or no introgression of nuclear genes, usually from native to invasive species. Molecular markers, which are subject to high interspecific gene flow, may be protected from introgression [22] and thus are theoretically more suitable for species identification [23].

However, molecular markers based on cytoplasmic genes or nuclear gene fragments contain limited genetic information, and the large genomes of *Picea* make resequencing too costly. Therefore, *P. pungens* and *P. jezoensis* were used as outgroups in this study. *P. wilsonii*, *P. neoveitchii* and the close relative *P. likiangensis* [18] were selected as the experimental materials. Genome-wide single nucleotide polymorphism (SNP) markers were obtained by genotyping-by-sequencing (GBS) technology to study the gene flow among the three spruce species and the genes under selection during species differentiation. To explain the phylogenetic relationships of these three species from the perspective of the whole genome, we revealed the function of the selected genes during species differentiation and provided a scientific basis for the introduction of *Picea*.

## 2. Results

### 2.1. Sequencing and Variant Discovery

The raw image data files obtained from GBS were transformed into raw sequences by base identification analysis. Sixty *Picea* samples were sequenced to obtain a total of 328.24 Gb of raw data, and the amount of clean data generated after removing the low-quality sequences was 317.10 Gb, with an average data volume of 5.28 Gb per sample. The quality of the sequenced bases was high (Q20 ≥ 97.83%, Q30 ≥ 93.32%), the GC content with a mean of 39.55% was distributed normally, and the average alignment rate between the population samples and the *P. abies* reference genome was 96.43%, indicating that the four spruce species were highly similar to *P. abies*. A total of 9,162,074 SNP loci were obtained after detection by GATK ver. 4.2.2.0 software (McKenna, MA, USA). After filtering under the conditions of a minor allele frequency (MAF) > 0.01 and maximum missing rate < 0.5, a total of 1,710,226 high-quality SNP loci were obtained for population analysis.

### 2.2. Phylogenetic and Population Structure Analyses

**Figure 1:**
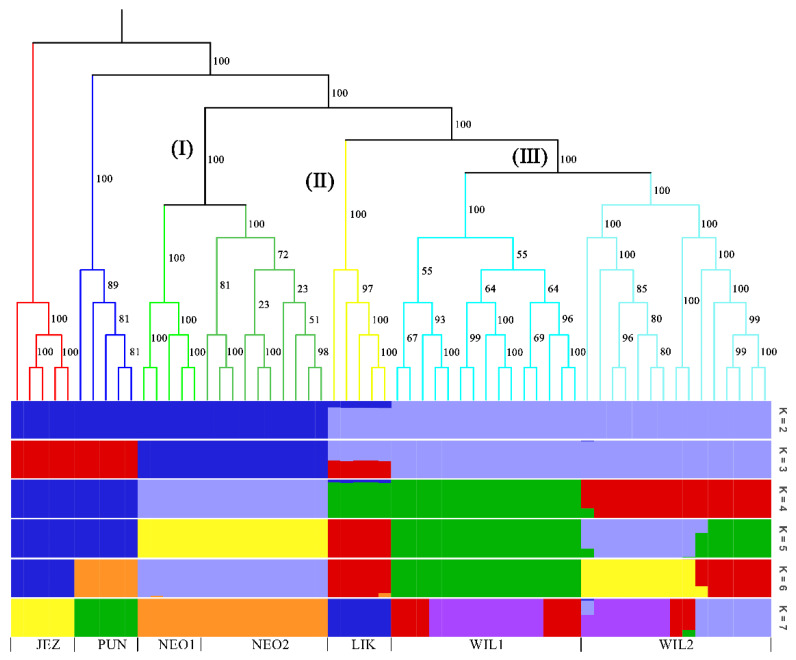
Maximum likelihood tree and structure analysis histogram of five spruce species based on SNP markers. JEZ, *P. jezoensis*; PUN, *P. pungens*; NEO1, *P. neoveitchii* from Gansu; NEO2, *P. neoveitchii* from Shaanxi; LIK, *P. likiangensis*; WIL1, *P. wilsonii* from Shanxi; WIL2, *P. wilsonii* from Hubei. The colors in the upper part of the figure represent different groups and the colors in the lower part of the figure represent different genetic components.

**Figure 2:**
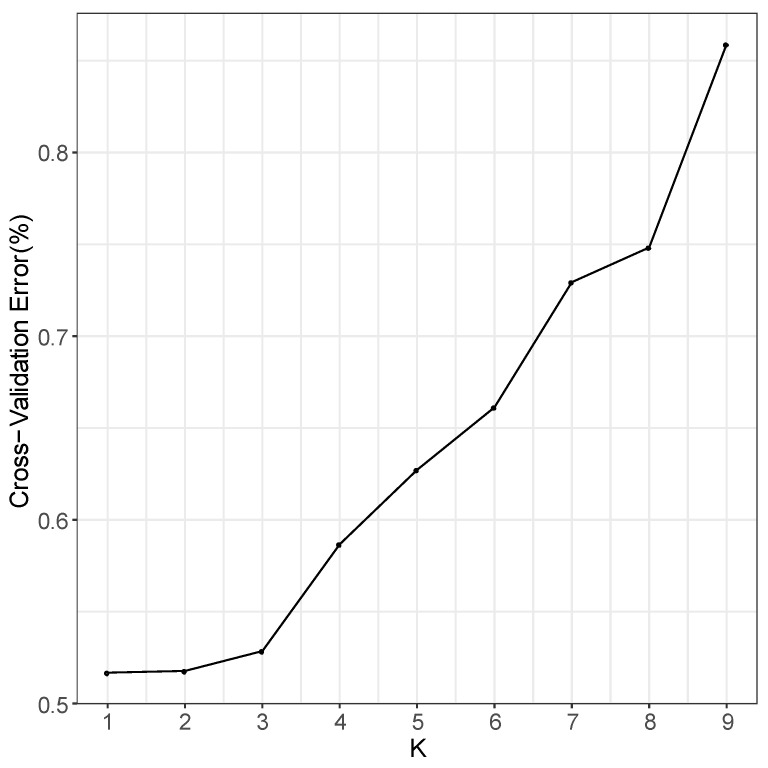
Cross-validation error value.

**Figure 3:**
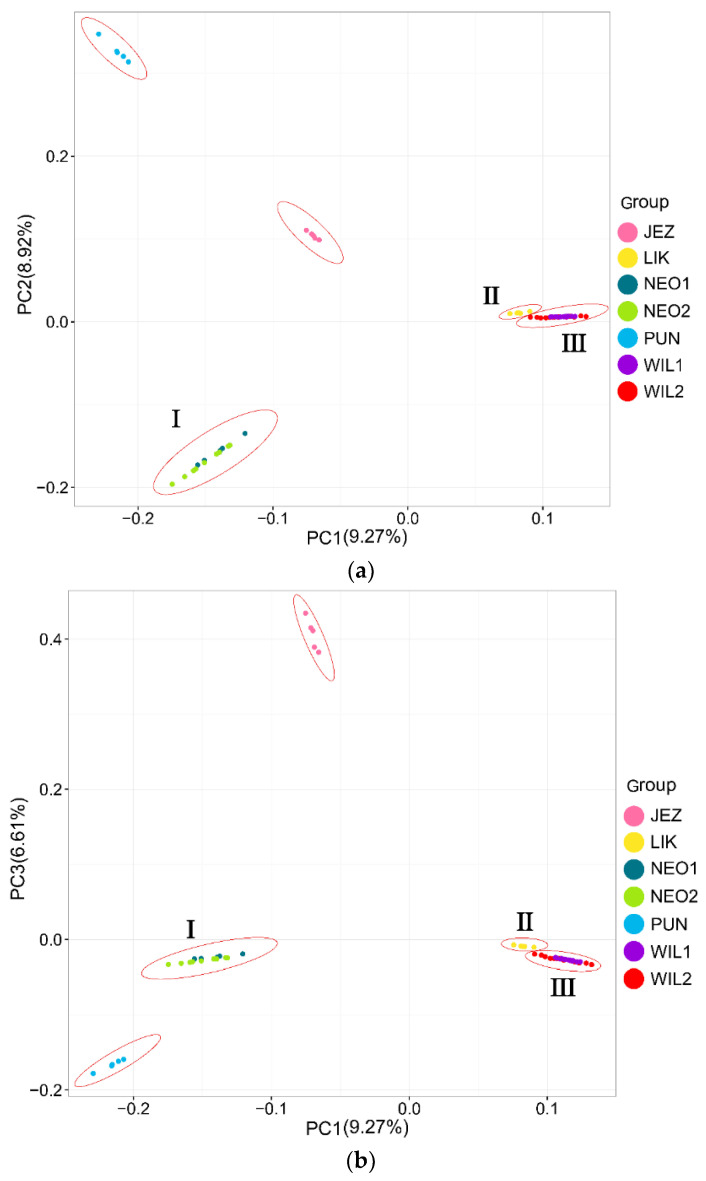
Principal component analysis of 60 spruce plants. (**a**) PC1 & PC2, (**b**) PC1 & PC3. The species abbreviations are the same as in Figure 1. I, II and III are the three subgroups in Figure 1.

In the phylogenetic tree constructed by the maximum likelihood (ML) method and using *P. jezoensis* and *P. pungens* as outgroups, shown in Figure 1, the 50 *Picea* individuals clustered into three main taxa (I–III). Among them, Group I consisted of two *P. neoveitchii* populations collected from Gansu and Shaanxi, Group II consisted of *P. likiangensis* from Yunnan, and Group III consisted of two *P. wilsonii* groups from Shanxi and Hubei. Admixture ver. 1.3.0 software (Alexander, CA, USA) was used to further evaluate the population structure with different K values (Figure 1). According to the cross-validation (CV) error values presented in Figure 2, the CV error was smaller when K = 2–7, indicating more reasonable results with 2–7 subgroups. When K = 2, two populations of *P. wilsonii* were separated from the other populations, while *P. likiangensis* had two genetic components. When K = 3, two populations of *P. neoveitchii* were separated from the *P. jezoensis* and *P. pungens* components to form a new component, and the population of *P. likiangensis* was composed of the *P. wilsonii* component and the *P. jezoensis* and *P. pungens* components. When K = 4, two populations of *P. wilsonii* were separated to form two new components. The population of *P. likiangensis* was composed of the component of *P. wilsonii* from Shanxi and the component of *P. jezoensis* and *P. pungens*. When K = 5, *P. likiangensis* separated to form a new component, and some individuals of *P. wilsonii* from Hubei clustered into one component with *P. wilsonii* from Shanxi, and the population structure was more consistent with the groups depicted in the phylogenetic tree. When K = 6, *P. jezoensis* and *P. pungens* were separated, and some individuals of *P. wilsonii* from Hubei, which had previously been clustered as a component with *P. wilsonii* from Shanxi, were clustered as a component with *P. likiangensis*. When K = 7, *P. wilsonii* from Shanxi separated to form two components, while some individuals of *P. wilsonii* from Hubei that had clustered with *P. likiangensis* into one component separated to form a new component, and the remaining individuals of *P. wilsonii* from Hubei clustered with some of those from Shanxi into one component. Therefore, *P. wilsonii* is more closely related to *P. likiangensis* than to *P. neoveitchii*, and there is some differentiation between *P. wilsonii* from Hubei and Shanxi, while there is less of a difference between the two populations of *P. neoveitchii*. To further analyse the population structure, principal component analysis (PCA) was used to reveal the relationships among the 60 spruce samples. As shown in Figure 3, because of the complex genetic background of the natural spruce populations and the large amount of variation, the three principal components explained little variation, but PCA also revealed three main groups: group I was *P. neoveitchii*, group II was *P. likiangensis* and group III was *P. wilsonii*. This is consistent with the grouping from the phylogenetic tree and population structure analysis, further validating the topology of the phylogenetic tree.

### 2.3. Gene Flow between Populations

**Figure 4:**
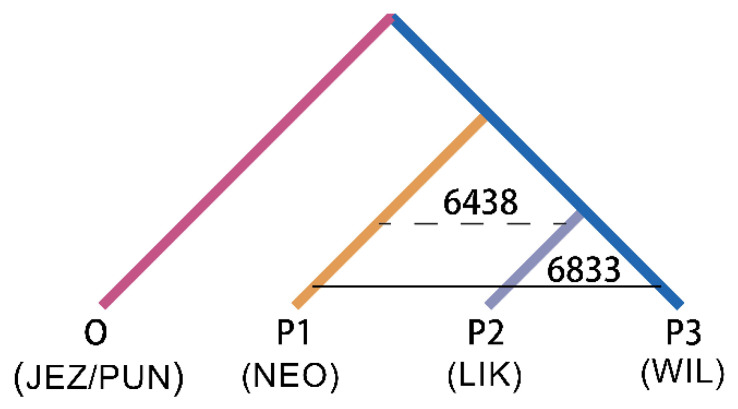
ABBA-BABA test tree model of five spruce species. NEO, *P. neoveitchii* from Gansu and Shaanxi; WIL1, *P. wilsonii* from Shanxi and Hubei; others are the same as in Figure 1.

Based on the tree topology shown in Figure 4, gene flow between species was further revealed using the ABBA-BABA test. When *P. neoveitchii* was the ancestral group and *P. wilsonii* and *P. likiangensis* were sister species, the ABBA-BABA test showed a significant positive value (D = 0.030, Z score > 3) (Table 1). A total of 6833 alleles were shared between *P. neoveitchii* and *P. wilsonii*, while 6438 alleles were shared between *P. neoveitchii* and *P. likiangensis*. Thus, there was unequal gene flow between *P. neoveitchii* and the two sister species. The gene flow rate between *P. neoveitchii* and *P. wilsonii* was greater than that between *P. neoveitchii* and *P. likiangensis*; that is, there was significant gene flow between *P. wilsonii* and *P. neoveitchii*.

### 2.4. Selective Sweeps Increased Genetic Differentiation between P. wilsonii, P. likiangensis and P. neoveitchii

Selective sweep analysis mainly detects the genes under selection in a population and further reveals the adaptive mechanism of population evolution. In this study, two populations of *P. neoveitchii* were used as background group A and two populations of *P. wilsonii* and *P. likiangensis* were used as test groups B and C, respectively. Nucleotide diversity (*π*) and population genetic difference (*F_st_*) were used in combination to identify regions where selective sweeps occurred during species differentiation. A total of 948 selected genes were identified in different subgroups in comparison with the *P. neoveitchii* background group, with a total of 326 genes between *P. wilsonii* and *P. neoveitchii* and 622 genes between *P. likiangensis* and *P. neoveitchii* (Tables S1 and S2).

**Figure 5:**
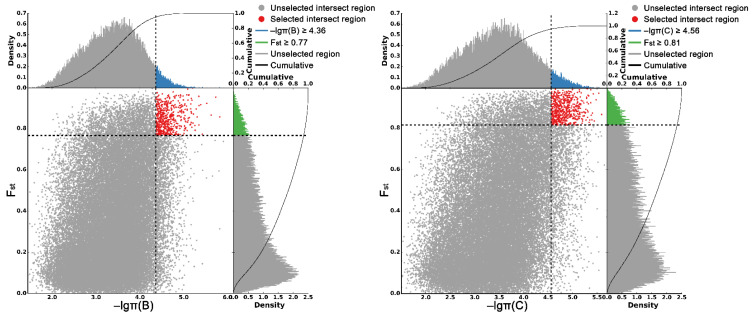
Genomic regions with selective sweep in *P. wilsonii* and *P. likiangensis*. B, *P. wilsonii*; C, *P. likiangensis*.

**Figure 6:**
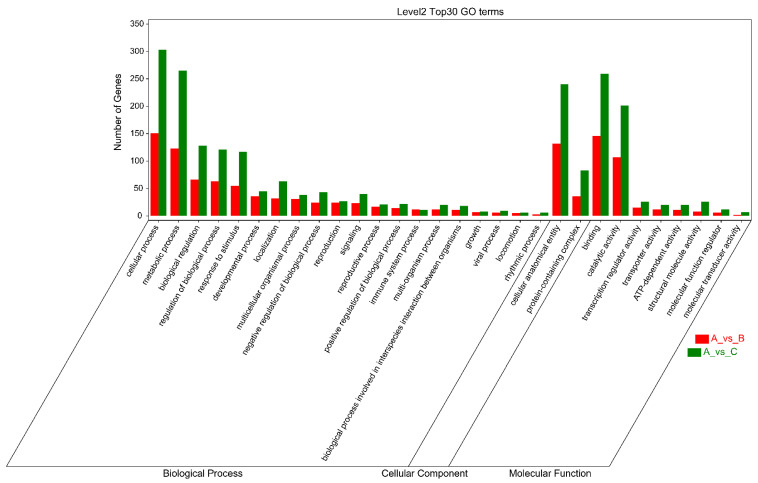
Cluster map for GO enrichment analysis of selected genes. A, *P. neoveitchi*; B, *P. wilsonii*; C, *P. likiangensis*.

**Figure 7:**
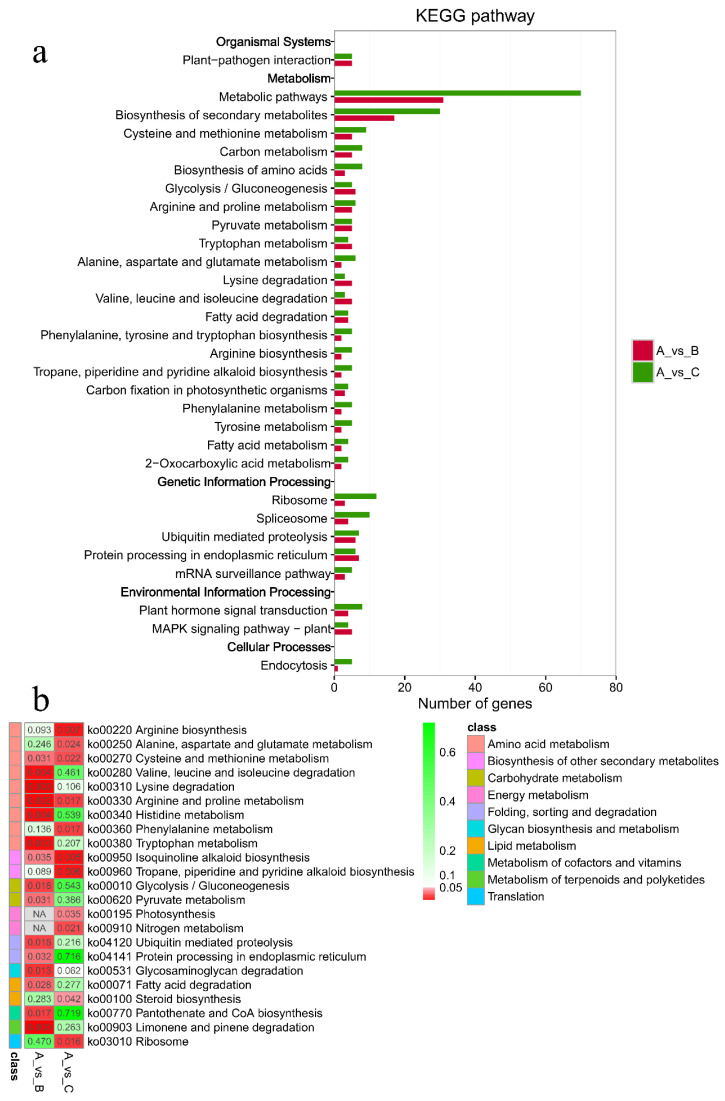
KEGG enrichment analysis of selected genes. (**a**) Hot map for significance of pathway. (**b**) Number of genes for pathway. A, *P. neoveitchii*; B, *P. wilsonii*; C, *P. likiangensis*.

As shown in Figure 5, the criteria for defining selective sweeps in *P. wilsonii*, using *P. neoveitchii* as the background group, were *F_st_*(B) ≥ 0.77 and *π*(B) ≤ 4.33 × 10^−5^ (−lg*π*(B) ≥ 4.36). Based on GO enrichment analysis, it was clear that the molecular functions of the selected genes were mainly concentrated in protein binding, nucleic acid binding, DNA-binding transcription factor activity and methyltransferase activity (Figure 6; Table S3). Pathway analysis was performed on the selected genes, and hypergeometric tests were applied to identify pathways that were significantly enriched among the genes detected in *P. wilsonii* compared to those in the entire genome of the background population of *P. neoveitchii*. Figure 7 shows that the pathways mainly related to lysine degradation, limonene and pinene degradation, and arginine and proline metabolism. Among the selected genes, genes associated with plant disease resistance (e.g., *BSK5* and *ERECTA*) and environmental stress (e.g., *ABCC2*, *CESA6*, *CUL*, *UPF3*, *VIR* and *ZIF1*) were identified, which may be associated with the wide distribution and environmental adaptability of *P. wilsonii*. Two genes associated with plant morphology (*Sb09* and *SRL1*), three genes associated with plant stomatal regulation (*KIN10*, *NPF4.6* and *SCRM2*) and six genes associated with flowering (*AIL5*, *AP2*, *NAP1*, *NPF8.3*, *PRMT6* and *ubp13*) were also identified within the selected regions. This suggests that the functions of the target genes subject to selection between *P. wilsonii* and *P. neoveitchii* were mainly plant stress tolerance, stomatal regulation and flowering.

*P. likiangensis* was subjected to selective sweep analysis in comparison with the background group of *P. neoveitchii*, with the criteria being *F_st_*(C) ≥ 0.81 and *π*(C) ≤ 2.75 × 10^−5^ (−lg*π*(C) ≥ 4.56) (Figure 5). GO enrichment analysis (Figure 6; Table S4) and pathway analysis (Figure 7) of the selected genes revealed that their molecular functions were mainly in protein binding, nucleic acid binding, DNA binding and RNA binding. The pathways were mainly concentrated in isoquinoline alkaloid biosynthesis, tropane, piperidine and pyridine alkaloid biosynthesis, arginine biosynthesis, and ribosomes. Three genes associated with flowering time (*ubp13*, *CRY1* and *sc35*) were found in selected areas of *P. likiangensis* in contrast to *P. neoveitchii*, which may be related to the adaptation of *P. likiangensis* to high-altitude environments. There were also 13 genes related to plant stress resistance (*RAV1*, *KPNB1*, *GT2*, *SRG1*, *NAC100*, *SGS3*, *AG1*, *EXLA2*, *ERECTA*, *CESA6*, *CYP86A2*, *GR* and *CYP74A*) in the selected region, indicating that *P. likiangensis* and *P. neoveitchii* were highly divergent in terms of plant stress tolerance. In addition, a gene related to leaf morphology, *SRL1*, and two genes associated with phosphorus regulation, *ALMT5* and *WRKY42*, were identified.

## 3. Discussion

### 3.1. Phylogeny and Gene Flow Characteristics

Recent radiative divergence and frequent interspecific introgression of *Picea* complicated the phylogenetic relationships within the genus. The Bayesian phylogenetic tree of *Picea* based on chloroplast, mitochondrial and nuclear gene fragments showed that *P. likiangensis* and *P. neoveitchii* evolved from a common ancestor, and their relationship was closer than that of either of them with *P. wilsonii* [17]. In contrast, an ML phylogenetic tree based on the whole genome and PCA in our study showed that *P. likiangensis* was more closely related to *P. wilsonii* than to the other species, which was consistent with the results of the phylogenetic tree constructed by Feng et al. [18] based on nuclear gene transcriptome data. Through the study of SNP site variation differentiation of nrDNA and pollen morphological variation, a natural hybrid between *P. likiangensis* and *P. wilsonii*, *P. purpurea*, was found [24,25]. Specifically, a Bayesian computing (ABC) method was used to detect the variation in nrDNA sequences, and it was found that *P. purpurea* was a natural hybrid of *P. likiangensis* and *P. wilsonii*, with 69% of its genetic components from *P. likiangensis* and 31% from *P. wilsonii* [24]. A study of pollen morphology revealed that the surface texture of the pollen of *P. purpurea* was consistent with that of *P. likiangensis*, and the morphologies of pollen sacs and pollen bodies were similar to those of *P. wilsonii*, so it was also presumed to be a hybrid of *P. likiangensis* and *P. wilsonii* [25]. However, a study based on nuclear gene transcriptome data suggested that *P. purpurea* was not a hybrid and found that *P. brachytyla* var. *brachytyla* may be a variety of hybrid origin. Part of its genetic composition is attributable to *P. wilsonii*, and the other part is closely related to *P. likiangensis*, *P. brachytyla* var. *complanata* and *P. farreri* [9]. These results further indicated that *P. likiangensis* was closely related to *P. wilsonii*, and there was frequent gene exchange between them. The cpDNA of *Picea* was paternally inherited, its mtDNA was maternally inherited and their DNA variation was more easily lost compared to that of nrDNA, while species-specific mutations accumulated at a faster rate, which tended to distort the true interspecific relationships. The genetic information contained in a single or multiple nuclear gene fragments is limited, and the study of Shen et al. [9] suggested that at least 600 nuclear genes were required to resolve the interspecific relationships of *Picea*. The small number of nuclear genes makes it difficult to fully and truly reflect the evolutionary information of species [26]. Our study better reflects the evolutionary relationships between the three species at the genome-wide level.

Although the divergence between *P. neoveitchii* and *P. wilsonii* occurred early, there was continuous mitochondrial gene introgression and little gene introgression [19] due to the overlapping distribution areas of *P. wilsonii* and *P. neoveitchii* in their natural environment. Moreover, there was some degree of nuclear gene introgression but less introgression in the chloroplast genome [21]. However, the genome-wide ABBA-BABA analysis performed in our study showed that the gene flow rate between *P. wilsonii* and *P. neoveitchii* was greater than that between *P. likiangensis* and *P. neoveitchii*, and there was also significant gene flow between *P. wilsonii* and *P. likiangensis*. Previous studies used nuclear gene fragments to analyse gene flow between *P. wilsonii* and *P. neoveitchii* [19], making it difficult to fully reflect the true extent of gene flow in the nuclear genome.

### 3.2. Selective Sweeps Contributing to Adaptation in Picea

Selective sweeps usually result in a reduction in the diversity of the locus and associated gene regions, and they facilitate the adaptation of species to their environment. Although the number of species in *Picea* is relatively small, the genus is distributed from a temperate continental climate at 23° N to a cold temperate climate at 53° N and alpine or subalpine landforms. Selective sweep analysis could reveal key gene regions associated with differentiation among species in *Picea*, furthering our understanding of spruce environmental adaptation mechanisms. In our study, *P. neoveitchii* was used as the background population, and the genes selectively swept from the background population in *P. wilsonii* and *P. likiangensis* were mainly associated with plant stress resistance, flowering and leaf morphology. In *Arabidopsis thaliana*, *AIL5* is involved in the regulation of inflorescence development in coordination with auxin [27], *ubp13* is involved in the biological clock and photoperiodic flowering regulation [28], *CESA6* is important for salt stress tolerance [29] and *ERECTA* controls developmental processes and disease resistance [30]. *NAP1* plays an important role in the flowering process of *Phyllostachys edulis* [31]. The population of *P. neoveitchii* is sparse, narrowly distributed and extremely strict in its habitat requirements. It is scattered in forests or rock crevices at 1300–2000 m [32], while *P. wilsonii* has strong adaptability and is widely distributed and *P. likiangensis* is distributed in the alpine zone of acidic mountain brown forest soil at an altitude of 2500–3800 metres [15]. *P. neoveitchii* has stricter requirements for site conditions than *P. wilsonii* and *P. likiangensis*. Therefore, the genes selected in *P. wilsonii* and *P. likiangensis* were mainly genes that improve plant stress resistance. At the same time, due to differences in distribution range and region, phenology also differs among these species, and the genes that control plant development and photoperiod are under selection. This is similar to research results for *Juglans sigillata* and *J. regia*; Li et al. [33] also found genes related to flowering time in the selective sweep region of *J. sigillata* and *J. regia*. In addition, a gene related to leaf morphology, *SRL1*, was also identified, and needle leaf morphology is an important morphological indicator to distinguish spruce species. The leaves of *P. neoveitchii* and *P. wilsonii* are quadrangular; the leaves of *P. likiangensis* are flat quadrangular [15]. The needles of *P. neoveitchii* are larger than those of both *P. wilsonii* and *P. likiangensis*, the smaller needles help *P. wilsonii* and *P. likiangensis* to adapt to higher latitudes or higher altitudes. Two genes related to phosphorus regulation were found in the selected area of *P. likiangensis*, which may be because the natural population of *P. likiangensis* grows in acidic mountain brown forest soil, where the content of unstable total phosphorus in the humus is high [34] and phosphorus is easily lost through leaching. For adaptation to the local environment, the functions of genes related to phosphorus regulation in *P. likiangensis* are enhanced and differentiated. Among the genes under differential selection between *P. wilsonii* and *P. neoveitchii*, genes related to stomatal regulation and a gene related to plant height, *Sb09*, were also found. Each opening and closing of the stomata affect the transpiration, photosynthesis, and respiration of the plant. When the stomata of the plant are opened, transpiration becomes stronger, thereby protecting the plant from UV damage. The variation in stomatal regulation of *P. wilsonii* has also further enhanced its adaptability to the external environment, thus giving it a wider distribution than those of *P. likiangensis* and *P. neoveitchii*. In addition, *P. wilsonii* also has a taller plant height than *P. neoveitchii*, which allows *P. wilsonii* to receive more light and become the dominant species in the community. With the uplift of the Tibetan Plateau and the oscillation of the Quaternary glaciation, the environmental adaptation of spruce species was enhanced through the selective sweep of rapidly fixed favourable mutant genes under the pressure of environmental change and thus promoted the differentiation of species within the genus.

## 4. Materials and Methods

### 4.1. Sample Collection and DNA Extraction

**Figure 8:**
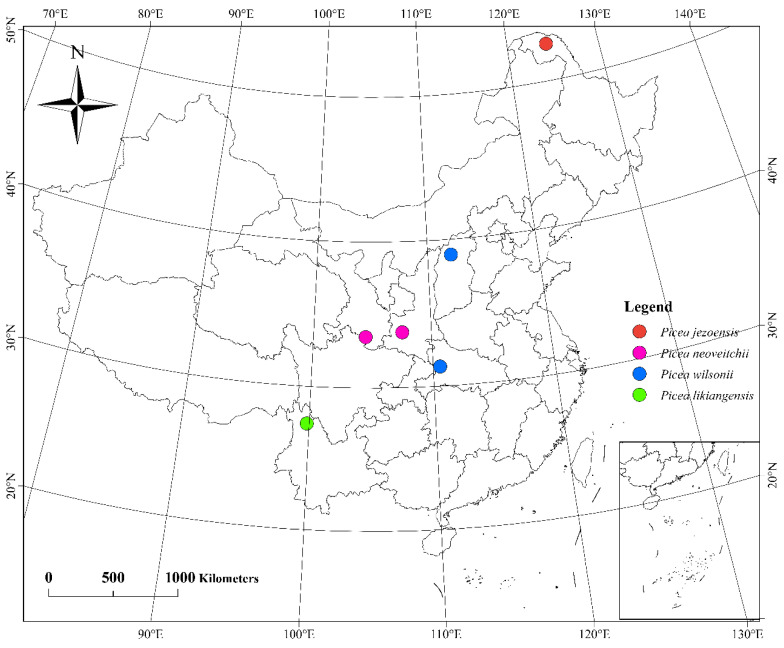
Sampling site map of 55 *Picea* plants in China.

Needle material was collected from a set of 60 *Picea* samples, including 15 trees of *P. wilsonii* from Hubei and Shanxi, 10 trees of *P. neoveitchii* from Shaanxi, 5 trees of *P. neoveitchii* from Gansu, 5 trees of *P. likiangensis* from Yunnan, 5 trees of *P. jezoensis* from Heilongjiang (Figure 8, Table S5) and 5 trees of *P. pungens* introduced from America. Samples were taken from trees at least 100 m apart in each population, and needles were dried directly with silica gel. The latitude, longitude and altitude of each sampling location were recorded using an eTrex GIS monitor (Garmin, Germany).

Silica-dried needles were used for DNA isolation with a modified cetyltrimethylammonium bromide (CTAB) extraction method [35]. A Nanodrop-1000 spectrophotometer (Nanodrop, MA, USA) was used to measure DNA purity (OD260/OD280), and 1% agarose gel electrophoresis was used to test DNA purity and integrity.

### 4.2. GBS and SNP Analysis

GBS was performed as described by Poland et al. [36,37] at Genedenovo Bioinformatics Technology Co., Ltd., Guangzhou, China. A total of 1.5 μg DNA from each sample was digested with EcoRI and NiaIII restriction enzymes in 96-well plates. The two ends of the digested DNA fragments were ligated with adapters using T4 DNA ligase, and then each sample was amplified. The 400–600 bp DNA fragments were isolated by electrophoresis for purification and sequenced on a HiSeq4000 instrument (Illumina, CA, USA).

For calling SNPs, raw data were processed to obtain clean data through the fastp application [38]. Adapter sequences and abnormal nucleotide bases at the 5′ terminus were removed from the raw data. Moreover, the low-quality ends of the reads were removed, including reads with ≥10% identified nucleotides (N) and reads with >50% bases having Phred quality scores of ≤10(S6). The remaining clean data were mapped to the European spruce reference genome using the Burrows—Wheeler Aligner (BWA) program ver. 0.7.8-r455 (Li, Cambridge, UK) with the command ‘mem-t4-k32-M’ [39]. After alignment, SNP calling on a population scale was performed using a Bayesian approach as implemented in the package GATK [40]. SNPs were filtered using GATK’s Variant Components with percent standards (Window, 4–filter “QD < 2.0 | | FS > 60.0 | | MQ < 40.0”, -G_filter “GQ < 20”). The resulting SNPs were then further filtered through VCFtools v.0.1.11 (Danecek, Cambridge, UK) [41], and only high-quality SNPs (MAF ≥ 0.01, maximum missing rate ≤ 0.5) were kept for phylogenetic analysis.

### 4.3. Phylogenetic, Population Structure and Principal Component Analyses

To reconstruct the phylogenetic relationships among species, a phylogenetic tree of 60 samples was constructed based on the SNP dataset using the ML method by IQTree v1.6.10 (Nguyen, Vienna, Austria) [42]. Bootstrap values were calculated 1000 times, and the GTR + I + G model was selected. The tree file was imported into the Interactive Tree of Life (ITOL) (https://itol.embl.de/itol.cgi (accessed on 30 August 2022)), which is an online tool for the display, annotation and management of phylogenetic trees.

Population structure cluster analysis based on ADMIXTURE ver. 1.3.0 software (Alexander, CA, USA) [43] was used for samples of *Picea* with K values ranging from 2 to 7. PLINK ver. 1.90 software (Purcell, MA, USA) [44] was used to convert the data file, and cross-validation was applied to explore convergence and determine the optimum number of clusters. The genetic component coefficient (Q) of each material in each subgroup was used to construct the population genetic structure matrix.

PCA was also used to evaluate the genetic structure of the spruce populations. The PCA of the SNPs was performed using the smartpca program in EIGENSOFT v5.0 (Patterson, MA, USA) [45], and the PCA distribution map was drawn by R ver. 4.2.1 software (Robert and Ross, Auckland, New Zealand).

### 4.4. Gene Flow and Introgression

To detect gene introgression among *P. wilsonii*, *P. neoveitchii* and *P. likiangensis*, we performed the ABBA-BABA test. The ABBA-BABA test, also known as the D-test, is used to detect whether the genetic composition of a population is affected by interspecific gene flow [46]. For the combination (((P1, P2), P3), outgroup) in the ABBA-BABA test, if the value of the D-test is positive, it means that P1 has the outgroup allele and P2 and P3 share the derived allele. The gene flow between P2 and P3 is greater than that between P1 and P3, that is, the ABBA pattern. If the D-test value is negative, it indicates that P1 and P3 share a derived allele, P2 has an outgroup allele and the gene flow between P1 and P3 is greater than that between P2 and P3, that is, the BABA pattern. Under the null hypothesis of incomplete lineage sorting (D = 0), the numbers of ABBA and BABA loci are expected to be equal. In this study, to avoid the effect of linkage disequilibrium between loci on ABBA-BABA detection, we first used PLINK ver. 1.90 software (Purcell, MA, USA) [44] to filter high-quality SNPs (indep-pairwise50 10 0.1). Then, we designated *P. pungens* and *P. jezoensis* as outgroups, *P. neoveitchii* as P1, *P. likiangensis* as P2 and *P. wilsonii* as P3 based on a phylogenetic tree constructed from genome-wide SNPs. A D-test analysis was performed using the Dtrios command of the Dsuite ver. 0.5-r44 software (Malinsky, Basel, Switzerland) package, calculated as D = (nABBA − nBABA)/(nABBA + nBABA) where nABBA is the total number of ABBA patterns and nBABA is the total number of BABA patterns [47]. To test for significance of the D-test, we used jackknife to calculate the z-score; when the absolute value of the z-score was greater than 3, the result was considered significant [46,48].

### 4.5. Genome-Wide Scan for Selection

The purpose of selective sweep analysis is to determine the selected region in the target population to further identify the selected genes and explain the species differentiation mechanism underlying population evolution at the genome-wide level. Selected regions are not only chromosomal regions with low genetic diversity but also regions with high rates of genetic differentiation among populations. Therefore, in this study, the selective scan region was determined by calculating population nucleotide diversity (*π*) and population genetic differentiation (*F_st_*) with VCFtools v.0.1.11 (Danecek, Cambridge, UK) [41]. Based on the results of *F_st_* and *π* at the top 5% threshold, significant regions were screened, and genes within the significant regions were extracted for pathway enrichment analysis. Pathway analysis based on the KEGG database can provide insight into the biological functions of genes and help screen genes enriched in metabolic pathways or signal transduction pathways.

## 5. Conclusions

In this study, we employed GBS technology to obtain genome-wide SNP markers and study the phylogenetic relationships, population structure, interspecific gene flow and selection pressure of *P. wilsonii*, *P. neoveitchii* and *P. likiangensis*. We found that *P. wilsonii* and *P. likiangensis* clustered onto one branch in the phylogenetic tree, and PCA also clustered them into one group, indicating that *P. wilsonii* and *P. likiangensis* are more closely related than the other pairs of species. Further ABBA-BABA analysis revealed that the gene flow rate between *P. wilsonii* and *P. neoveitchii* was greater than that between *P. likiangensis* and *P. neoveitchii*, probably because of the homozygous distribution of these two species. In the background population of *P. neoveitchii*, the genes that were selected in *P. wilsonii* were mainly related to plant stress resistance, stomatal regulation, plant morphology and flowering, while the genes selected in *P. likiangensis* were mainly related to plant stress resistance, leaf morphology and flowering time. The key functional genes under differential selection among spruce species were involved in improving species resistance, promoting flowering and fruiting ability, facilitating adaptation to different habitats, and continually enhancing the reproduction of breeding to advance evolution.

## Figures and Tables

**Table 1:** ABBA-BABA test of five spruce species.

P1	P2	P3	ABBA	BABA	D	Z-score
NEO	LIK	WIL	6438	6833	0.030	8.8916

The species abbreviations are the same as in Figure 4.

## Data Availability

The raw sequencing data generated from this study have been deposited in NCBI SRA (https://www.ncbi.nlm.nih.gov/sra (accessed on 3 September 2022)) under the accession number PRJNA876367.

## Supplementary Materials

The following supporting information can be downloaded at: https://www.mdpi.com/article/10.3390/plants11212993/s1, Table S1: Selected genes between *P. wilsonii* and *P. neoveitchii*; Table S2: Selected genes between *P. likiangensis* and *P. neoveitchii*; Table S3: GO enrichment (molecular function) analysis of selected genes between *P. wilsonii* and *P. neoveitchii*; Table S4: GO enrichment (molecular function) analysis of selected genes between *P. likiangensis* and *P. neoveitchii*; Table S5: Geographic information of the *Picea* populations in the study; Table S6: Statistics of nucleobase information before and after filtering.
